# Case Report: an unusual orbital tumor

**DOI:** 10.12688/f1000research.130056.2

**Published:** 2023-10-27

**Authors:** Anis Mahmoud, Hager Touil, Fadima Hann, Riadh Messaoud

**Affiliations:** 1 Department of Ophthalmology, Tahar Sfar University Hospital, Mahdia, 5100, Tunisia, Faculty of Medicine, University of Monastir, Monastir, Tunisia, Mahdia, Tunisia; 2 Department of Oral and Maxillofacial surgery, Tahar Sfar University Hospital, Mahdia, 5100, Tunisia, Faculty of Medicine, University of Monastir, Monastir, Tunisia, Mahdia, Tunisia

**Keywords:** Lipoma, Orbit, Histopathology, MRI, Surgery.

## Abstract

Introduction

Orbital lipoma is an extremely rare tumor, representing less than 1% of all orbital tumors. We review the literature and describe the presentation, the differential diagnosis and the management of this tumor.

Case report

We report the case of a 63-year-old patient who was referred for a diplopia with recent hemi-cranial headache. Physical examination showed no exophthalmos nor decrease in visual acuity. The patient complained of diplopia on elevation and oculomotricity examination showed limited elevation of the right eye. The Hess Lancaster test was in favor of a limited course of the right inferior rectus muscle. Magnetic resonance imaging revealed a fusiform tissue process in the right inferior rectus muscle with a fatty signal. A complete excision of the tumor was performed by a trasncunjonctival approach. Cytopathological examination was consistent with a pleomorphic lipoma. The postoperative period was uneventful. The definitive histopathologic diagnosis was a lipoma. The postoperative Magnetic resonance imaging showed the complete disappearance of the lesion. With 3 years of follow up, there is no sign of recurrence or ocular motility trouble.

**Conclusion:** Lipomas are rare tumors in the orbit. The clinic is variable depending on the size and the site. The clinical diagnosis is difficult to make. Only histology allows the final diagnosis.

## Introduction

Lipomas are benign tumors, rare in the orbit, representing less than 1% of all orbital tumors. They pose a differential diagnosis with a variety of other expansive orbital masses. ^1^ We report a new case, review the literature and discuss the clinicopathological and radiological features, the differential diagnosis and the management of this entity.

## Case report

**Figure 1.**
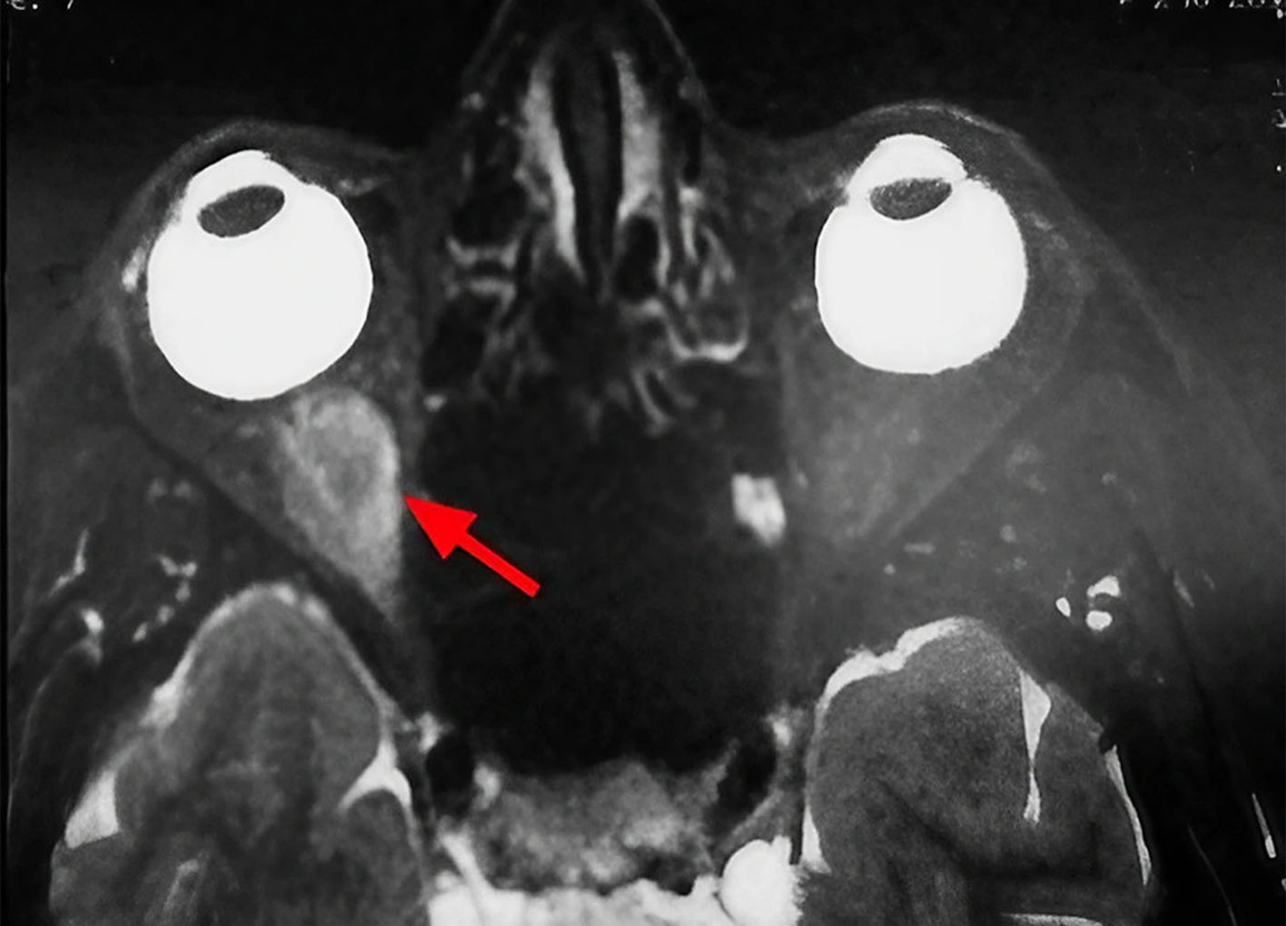
Sagittal section of orbital MRI showing a mass with high signal on a T2 weighted image (red arrow).

**Figure 2.**
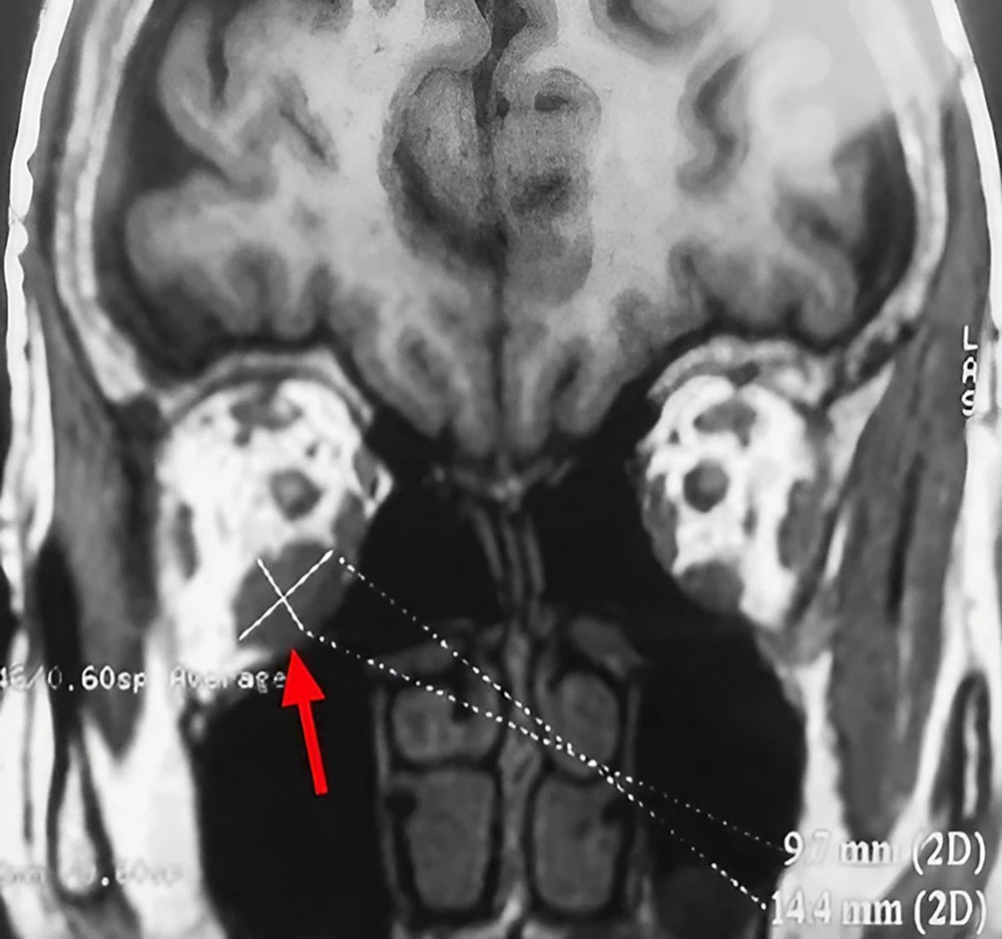
Coronal section of orbital MRI showing a mass with low signal on a T1 weighted image (red arrow).

A 63-year-old unemployed Tunisian woman, with no previous personal or family pathological history, presented with a diplopia with recent hemicranial headache evolving for two weeks. Physical examination showed no exophthalmia and no decrease in visual acuity. Furthermore, it revealed bilateral diplopia on elevation. Oculomotricity examination showed limited elevation of the right eye, which was confirmed by the Hess Lancaster test that revealed a limited course of the right inferior rectus muscle. General examination revealed no other lipoma locations. Magnetic resonance imaging (MRI) showed a fusiform and hyper-vascularized tissue process located in the right inferior rectus with fatty signal. The tumor was hyperintense on spin-echo T2-weighted images ( Figure 1) and hypointense on spin-echo T1-weighted images ( Figure 2).

These findings suggested various diagnosis; lipoma, inflammatory process, lymphoma and malignant tumor.

**Figure 3.**
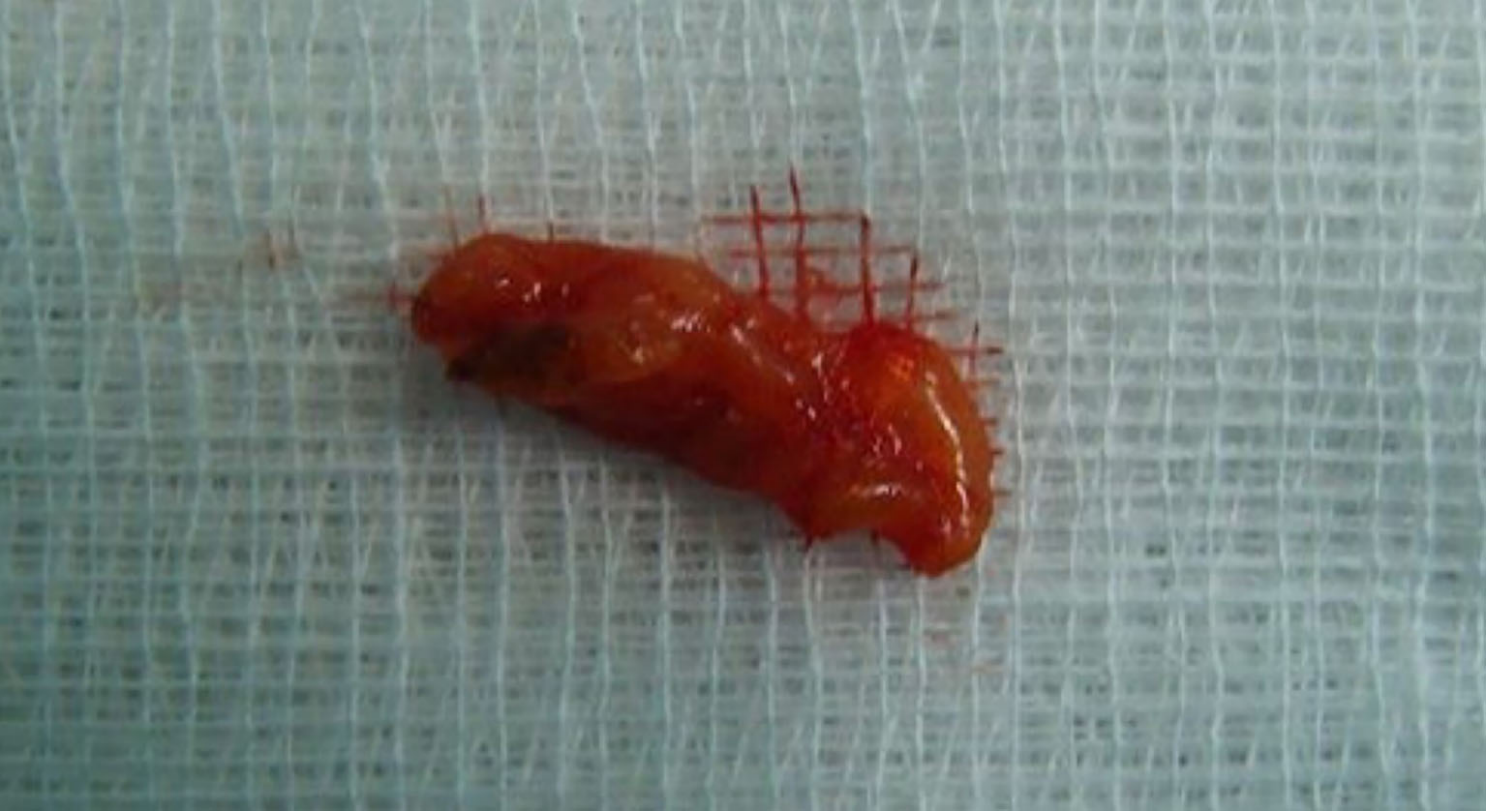
Macroscopic appearance of the specimen.

We performed a right inferior transconjunctival orbitotomy and excisional biopsy under general anesthesia. Peroperatively, we discovered an encapsulated mass of fatty tissue, thus complete excision was made. No adherences or involvement of adjacent structures occurred. The specimen was well circumscribed and slightly firmer than normal adipose tissue, with a yellow surface ( Figure 3).

**Figure 4.**
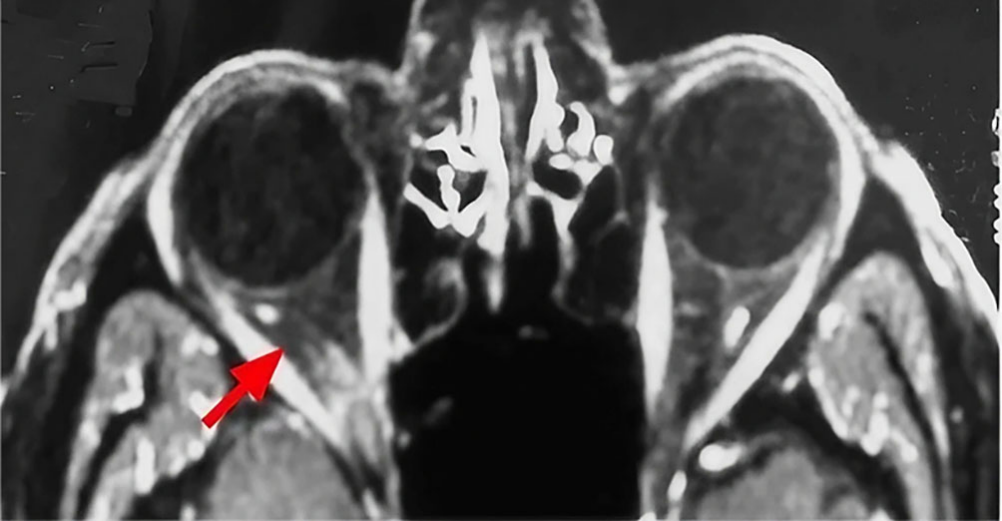
Postoperative axial MRI image demonstrating complete tumor resolution (red arrow).

Histologic examination was consistent with a pleomorphic lipoma. The postoperative period was uneventful. Immediately after the operation, the patient reports the resolution of his diplopia. Postoperative MRI images demonstrated the complete resolution of the tumor ( Figure 4). With 3 years of follow up, there is no sign of recurrence or ocular motility impairment.

## Discussion

Orbital lipoma is the most common mesenchymal soft tissue tumor. However, it is rarely found in the orbit despite the presence of abundant adipose tissue in the intraorbital space. ^2^^,^^3^ A review of the largest series of orbital tumors revealed a very low incidence of lipomas. ^4^ Shields *et al*. reported only two cases of lipomas in a review of 1264 cases of orbital tumors, indicating the rarity of this entity. ^5^ On physical examination, the diagnosis is often difficult to suggest. These tumors are often asymptomatic.

However, they can cause severe morbidity by causing progressive and painless exophthalmos, which is occasionally coupled with diplopia or ocular motility defects ^6^ such as was observed in our patient.

Orbital lipoma exceptionally leads to a compressive neuropathy responsible for a significant decrease in visual acuity, an alteration of the afferent photomotor reflex and the visual field constriction. ^1^ Imaging based on computed tomography (CT) scanning and MRI is essentially useful in ascertaining determining the exact seat, size and relationship to the orbit content. The fatty signal is characteristic on CT sequences. Furthermore, as was found in our patient, the tumor is hypointense on spin-echo T1-weighted images and hyperintense on spin-echo T2-weighted images. ^7^

Histology is essential for definitive diagnosis of pleomorphic lipoma. An important histologic criterion is the presence of a mixture of fat cells, pleomorphic cells and in particular floret-like multinucleated giant cells embedded in a myxoid stroma. ^8^ That concorded with the histological result of our case. Differential diagnosis of this tumor became more important because the number of reports about some other tumors of similar morphology, are increasing. Pleomorphic lipoma may be confused with lipomatous hemangiopericytoma, myofibroblastoma or even malignant tumors such as rhabdomyosarcoma, myxoid malignant fibrous histiocytoma and liposarcoma. ^9^^,^^10^ Surgical excision of an orbital lipoma is not only recommended for symptomatic cases such as our patient's clinical presentation but also to exclude malignancy. ^11^ In addition, as was noted in our patient, the long-term outcome after surgery is considered excellent. ^12^

This case highlights the importance of orbital imaging in the context of diplopia without obvious cause to rule out an intraorbital lipoma. Nevertheless, this association remains rare and requires further documentation of cases.

## Conclusion

Lipomas are benign soft tissue tumors, rarely located in the orbit. The clinical presentation is variable depends on the size and the intraorbital site. The histology makes the definitive diagnosis and may precisely identify the variant.

## Consent

Written informed consent for publication of their clinical details and/or clinical images was obtained from the patient.

## Data Availability

All data underlying the results are available as part of the article and no additional source data are required.
