# Supplementary material for: Case Report: an unusual orbital tumor
Source: F1000Res. 2023 Oct 27;12:700. Originally published 2023 Jun 19. [Version 2] doi: 10.12688/f1000research.130056.2 (PMC11079586; doi:10.12688/f1000research.130056.2)
Supplement: Peer review history

# Reviewer report: Reviewer response for version 2

**DOI:** 10.5256/f1000research.158021.r263307

**Published:** 2024-05-08

**Authors:** Kirthi Koka

**Affiliations:** 1 Orbit, Oculoplasty, Reconstructive and Aesthetic Services, Sankara Nethralaya Medical Research Foundation, Chennai, Tamil Nadu, India

The authors have presented an interesting case of orbital lipoma. 

 The authors have reviewed literature but there is no mention of the different variants of lipomas and how one can differentiate between these.

 The authors have added imaging features on MRI which is suggestive of hypointense lesion on T 1 weighted images.

 But as per most literature, Lipomas being predominantly made up of fat, are hyperintense on T1 weighted image ( as also documented in one of the references cited in the article - reference no 12). This does not corelate with the imaging findings and descriptions. Kindly clarify the same

 Also as per the imaging findings the lesion seems to be infiltrating into the inferior rectus muscle. The authors need to elaborate the surgical findings on table, if muscle fibres were involved or if the lesion was a well encapsulated, separate lesion that could be easily dissected and removed with no damage to the muscle fibres.

 In discussion the authors mention that imaging helps confirm the exact seat. Kindly elaborate what they mean by seat.

Are enough details provided of any physical examination and diagnostic tests, treatment given and outcomes?

Partly

Is the case presented with sufficient detail to be useful for other practitioners?

Yes

Is sufficient discussion included of the importance of the findings and their relevance to future understanding of disease processes, diagnosis or treatment?

Partly

Is the background of the case’s history and progression described in sufficient detail?

Yes

Reviewer Expertise:

Orbit and Oculoplasty

I confirm that I have read this submission and believe that I have an appropriate level of expertise to confirm that it is of an acceptable scientific standard, however I have significant reservations, as outlined above.

# Reviewer report: Reviewer response for version 1

**DOI:** 10.5256/f1000research.142784.r193958

**Published:** 2023-10-16

**Authors:** Amira Trigui

**Affiliations:** 1 Department of Ophthalmology, University of Sfax, Sfax, Tunisia

A 63-year-old man presented with diplopia for two weeks. MRI of the brain and orbit revealed a process in the orbit with a fatty signal. Excisional biopsy of the mass resolved the diplopia, and histology revealed a pleomorphic lipoma. This report may help clinicians in the future to be alert to the possibility of the presence of an orbital tumor despite the absence of exophthalmos, which is a common finding in this setting. In this case, the clinical observation is detailed and the MRI images are of excellent quality, but a few points need to be clarified:

- Did the patient's general examination reveal obesity, or other lipoma locations?
- Did the authors perform orbital ultrasound before MRI imaging?
- Authors should specify whether diplopia is monocular or binocular.
- It would also be interesting if the authors could explain the absence of exophthalmos in this case.

Are enough details provided of any physical examination and diagnostic tests, treatment given and outcomes?

Partly

Is the case presented with sufficient detail to be useful for other practitioners?

Yes

Is sufficient discussion included of the importance of the findings and their relevance to future understanding of disease processes, diagnosis or treatment?

Yes

Is the background of the case’s history and progression described in sufficient detail?

Yes

Reviewer Expertise:

Ophthamology

I confirm that I have read this submission and believe that I have an appropriate level of expertise to confirm that it is of an acceptable scientific standard.

# Reviewer report: Reviewer response for version 1

**DOI:** 10.5256/f1000research.142784.r190823

**Published:** 2023-08-04

**Authors:** Mehdi Hasnaoui

**Affiliations:** 1 Department of Otolaryngology-Head and Neck Surgery, Tahar Sfar Hospital, Mahdia, Tunisia

**Editorial Note from F1000Research – 14 August 2023:** A COI statement has been added detailing a shared affiliation between the authors and reviewer, which was not declared at the time of the publishing of this report. The COI statement is below.

 ---

 This article is well written. It is easy to read. The figures are demonstrative. The authors report a rare case of an orbital lipoma revealed by isolated diplopia. The MRI was not very suggestive of a lipoma. The authors insisted on the interest of MRI in the context of diplopia. They discussed the clinicopathologic and radiological characteristics and the differential diagnosis of this entity.

 I have some minor comments:

**Abstract :**

- The authors state: “ *We report the case of a 63-year-old patient who was referred for a diplopia with recent **hemi-cranial headache***”. This symptom “ *hemi-cranial headache*” was not mentioned and written in the case report section.
- The authors state: “ ***Cytopathological*** *examination was consistent with a pleomorphic lipoma. The postoperative period was uneventful. The definitive histopathologic diagnosis was a lipoma*. ” Did the authors do a cytopathological examination and then a definitive histological examination? This examination “cytopatholgical” was not mentioned and written in the case report section.

- Line 13 : change "trasncunjonctival" to "transcunjonctival"

**Case report : **

- The authors state: “ *MRI showed a spindle-shaped and hyper-vascularized tissue process*”. How to explain this hypervascularization?
- Do the authors have postgadolinium fat-saturated axial T1 images? Lipomas are classically hypointense after fat suppression.

**Discussion :**

-  In this case, the tumor was hyperintense on spin-echo T1-weighted images. This is not usual in lipomas and should be discussed.
- The authors state: “Furthermore, as was found in our patient, the tumor is hypointense on spin-echo T1-weighted images and hyperintense on spin-echo T2-weighted images. ^1 ^” However, in the literature and in the cited reference number 1, most lipomas are generally hyperintense on T1-weighted imaging.

I believe the paper has academic merit, but minor revision is needed.

Are enough details provided of any physical examination and diagnostic tests, treatment given and outcomes?

Yes

Is the case presented with sufficient detail to be useful for other practitioners?

Yes

Is sufficient discussion included of the importance of the findings and their relevance to future understanding of disease processes, diagnosis or treatment?

Partly

Is the background of the case’s history and progression described in sufficient detail?

Yes

Reviewer Expertise:

Oncology, sinus and nasal cavity surgery, facial bone imaging

I confirm that I have read this submission and believe that I have an appropriate level of expertise to confirm that it is of an acceptable scientific standard, however I have significant reservations, as outlined above.

# Response

**Published:** 2023-08-04

**Authors:** Anis Mahmoud

**Affiliations:** Department of Ophthalmology, Tahar Sfar University Hospital, Tunisia

I would like to thank the reviewer for his interest in this work and will answer his queries in this report:

 The MRI appearance of pleomorphic lipomas is variable and not pathognomonic for diagnosis, as the variation in the ratio of adipose to non-adipose components results in a wide spectrum of imaging features. MRI features such as hyper-vascularized tissue process and hypointense T1 and hyperintense T2 character can be explained by the presence of a high proportion of non-adipose elements in pleomorphic lipomas.

 We have added a new reference (7) that also confirms our MRI results, which show the tumor to be hypointense in T1 and hyperintense in T2.

 The expression "trasncunjonctival" has been replaced by "transconjunctival".

 Cytopathological" has been replaced by "histopathological".

 The symptom "hemi-cranial headache" has been added to the "case report" section.
